# Bamboo Vinegar Decreases Inflammatory Mediator Expression and NLRP3 Inflammasome Activation by Inhibiting Reactive Oxygen Species Generation and Protein Kinase C-α/δ Activation

**DOI:** 10.1371/journal.pone.0075738

**Published:** 2013-10-04

**Authors:** Chen-Lung Ho, Chai-Yi Lin, Shuk-Man Ka, Ann Chen, Yu-Ling Tasi, May-Lan Liu, Yi-Chich Chiu, Kuo-Feng Hua

**Affiliations:** 1 Division of Wood Cellulose, Taiwan Forestry Research Institute, Taipei, Taiwan; 2 Department of Biotechnology and Animal Science, National Ilan University, Ilan, Taiwan; 3 Graduate Institute of Aerospace and Undersea Medicine, National Defense Medical Center, Taipei, Taiwan; 4 Department of Pathology, Tri-Service General Hospital, National Defense Medical Center, Taipei, Taiwan; 5 Graduate Institute of Life Science, National Defense Medical Center, Taipei, Taiwan; 6 Department of Nutritional Science, Toko University, Chiayi, Taiwan; 7 Department of Biomechatronic Engineering, National Ilan University, Ilan, Taiwan; University of California Merced, United States of America

## Abstract

Bamboo vinegar (BV), a natural liquid derived from the condensation produced during bamboo charcoal production, has been used in agriculture and as a food additive, but its application to immune modulation has not been reported. Here, we demonstrated that BV has anti-inflammatory activities both *in* *vitro* and *in* *vivo*. BV reduced inducible nitric oxide synthase expression and nitric oxide levels in, and interleukin-6 secretion by, lipopolysaccharide-activated macrophages without affecting tumor necrosis factor-α secretion and cyclooxygenase-2 expression. The mechanism for the anti-inflammatory effect of BV involved decreased reactive oxygen species production and protein kinase C-α/δ activation. Furthermore, creosol (2-methoxy-4-methylphenol) was indentified as the major anti-inflammatory compound in BV. Impaired cytokine expression and NLR family, pyrin domain-containing 3 (NLRP3) inflammasome activation was seen in mice treated with creosol. These findings provide insights into how BV regulates inflammation and suggest that it may be a new source for the development of anti-inflammatory agents or a healthy supplement for preventing and ameliorating inflammation- and NLRP3 inflammasome-related diseases, including metabolic syndrome.

## Introduction

BV is a natural liquid derived from the condensed vapor produced during bamboo charcoal production. It is composed of water (∼80–90%), acetic acid, and many other organic constituents, and has a sour and smoky odor and a pH of 2.5 to 2.8 [1]. Addition of BV to pig manure undergoing composting reduces total Kjeldahl nitrogen loss and controls Cu and Zn mobility [2]. Feeding of chickens with a commercial diet supplemented with silicic acid and BV liquid leads to increased body weight gain by stimulating absorption by intestinal epithelial cells [3]. BV has germicidal activity against encephalomyocarditis virus, but this effect is lost when BV is brought to neutral pH [4]. Although BV has been used in food processing and as a food additive and shows medical properties, including the ability to eliminate toxins from the human body, no anti-inflammatory activity of BV has been previously explored.

The innate immune system in mammals is triggered by pathogen-associated molecular patterns that are recognized by toll-like receptors (TLRs) expressed on the surface of macrophages [5]. Lipopolysaccharide (LPS), a major cell wall component of Gram-negative bacteria, activates macrophages by binding to TLR4, and these results in activation of mitogen-activated protein kinases (MAPK), nuclear transcription factor kappa-B (NF-κB), or protein kinase C (PKC), which leads to the production of pro-inflammatory mediators [6]. Interleukin-1β (IL-1β) is one of the important cytokines produced by activated macrophages and its release is controlled by the inflammasome, a caspase-1-containing multi-protein complex [7], [8]. The most thoroughly characterized inflammasome is the NLRP3 inflammasome [9], [10], which controls the inflammatory responses caused by pathogen infection [11], [12], metabolic diseases [13]–[15], beta-amyloid [16], and silica crystals and aluminum salts [17]. Full activation of the NLRP3 inflammasome requires both a priming signal as a result of stimulation of pathogen recognition receptors, e.g., TLR4, which controls the expression of NLRP3 and IL-1β precursor, and an activation signal in response to a second stimulus, e.g. adenosine triphosphate (ATP), which controls caspase-1 activation [18], [19]. Reactive oxygen species (ROS) play a role in inflammatory cytokine production in response to LPS [20] and have also been shown to play an important role in NLRP3 inflammasome activation [21]. Inhibition of ROS production reduces NLRP3 inflammasome activation [19], [22]. Inhibition of inflammation and the NLRP3 inflammasome might prevent and ameliorate progression of diseases, including atherosclerosis [14], Alzheimer’s disease [16], gout [13], kidney disease [23], type 2 diabetes mellitus [24], and cancer [25].

Recently, the use of BV in the treatment of breast cancer and dermatitis has been patented, but no scientific papers of its use in medical applications have yet been published. Here, we demonstrate that BV reduces inflammatory mediator expression in LPS-activated macrophages. We further identified creosol as the major active compound of BV and suggested that it might prevent the progression of inflammation-related diseases.

## Materials and Methods

### Materials

LPS (from *Escherichia coli* 0111:B4), ATP, and mouse antibodies against mouse phospho-ERK1/2, phospho-JNK1/2, phospho-p38, actin and the chemicals without indicated were purchased from Sigma (St. Louis, MO). Rabbit antibodies against mouse phospho-PKC-α,* phospho-PKC-*δ,* IL-1*β, caspase-1, phospho-AKT, inducible nitric oxide synthase (iNOS), and cyclooxygenase-2 (COX-2), and horseradish peroxidase-labeled second antibodies were obtained from Santa Cruz Biotechnology (Santa Cruz, CA). IL-1β, interleukin-6 (IL-6), and tumor necrosis factor-α (TNF-α) ELISA kits were purchased from R&D Systems (Minneapolis, MN). Mouse anti-mouse NLRP3 antibody was purchased from Enzo Life Sciences Inc. (Exeter, UK). The AlamarBlue® assay kit was purchased from AbD Serotec Ltd (Oxford, UK) and the QUANTI-Blue™ reagent from InvivoGen (San Diego, CA).

### BV Preparation and Analysis

Moso bamboo (*Phyllostachys pubescens*) was provided by Pu Yuan Co. Ltd., Nantou, Taiwan. The samples were either steamed or heated (pre-processing), as in previous reports [26]–[28]. *These two types of treated bamboo were air-dried and specimens measuring 25* *mm×25* *mm×3* *mm (length×width×thickness) were prepared. All specimens were equilibrated at 20*°*C and 65*% *relative humidity for about four weeks, then the average moisture content and density were measured.*

The vinegar samples were provided by the Division of Forest Utilization, TFRI Taipei, Taiwan, and were collected at temperatures ranging from 90°C to 150°C based on the temperature measured by a thermocouple at the exit of the smoke funnel of the furnace during the bamboo charcoal manufacturing process [29]. The different BV samples were collected at 90–92°C (BV-1), 99–102°C (BV-2), 120–123°C (BV-3), and 145–150°C (BV-4) and were neutralized to pH 7 using NaOH for further experiments.

Fractionation was performed as described previously [30]. All mixing/extraction steps were for ten minutes at room temperature. Fifty grams of BV-4 (approximately 50 ml) was mixed with 200 ml of saturated salt solution, then the precipitate formed was removed by centrifugation and the supernatant again mixed as above with saturated salt solution, the precipitate removed and the supernatant treated again as above. The final supernatant was then extracted with 200 ml of ethyl ether. The ethyl ether phase was then mixed with 200 ml of 5% NaHCO_3_ solution to produce an ethyl ether phase and an aqueous phase. This aqueous phase was mixed with 50 ml of 30% H_2_SO_4_ and 200 ml of ether to generate an ether phase and an aqueous phase; the aqueous layer was dried down (acidic fraction), while the ether phase was mixed with 200 ml of 2N NaOH solution, yielding an ether phase containing neutral organic substances (neutral fraction), which was dried down, and an aqueous phase, which was mixed with 50 ml of 30% H_2_SO_4_ and 200 ml of ether and the aqueous layer containing phenolic substances dried down (phenol fraction).

Gas chromatography (GC) analysis was performed using a Shimadzu Hicap CBP20-M25 column (0.25 mm i.d.×25 m PEG-20M), a temperature of 60–200°C increasing at 5°C/min, then held at 200°C for 22 min, a splitter ratio of 60∶ 1, helium carrier gas, and flame ionization detection. Components were identified by comparing the retention times of their peaks with those of authentic compounds and those in the literature [30], [31]. Quantification of the components was based on peak areas expressed as a percentage of the total peak area. The amount of the phenolic, acidic, and neutral fractions injected was 1 µl of a 5% (v/v) solution in diethyl ether. Gas chromatography-mass spectrometry (*GC-MS*) was performed on a Jeol BU20 GC-Mate system. A GC equipped with a DB-WAX capillary column was connected directly to the mass spectrometer. The analytical conditions for GC were the same as those stated above. The operating conditions for the mass spectrometer were an ionization voltage of 70 *eV and an ionization source temperature of 250°C. Identification of the peaks was based on published mass spectrometry (MS) spectra data* [32]. *The phenol fraction was purified by high performance liquid chromatography (HPLC) (Column: ZORBAX Eclipse XDB-C18 column 4.6×150* *mm, 5* µ*m, mobile phase: ACN/H_2_O = 30/70, flow rate: 1* *ml/min). The pure creosol compound was obtained (t_R_ = 9.80* *min). The structure of the creosol was identified by nuclear magnetic resonance (NMR) and its character being described as follows: ^1^H NMR (CDCl3) δ2.21 (3H, s), 3.74 (3H, s), 5.84 (1H, s), 6.61 (1H, d, J = 8.7 Hz), 6.62 (1H,s), 6.80 (1H, d, J = 8.7 Hz); ^13^C NMR (CDCl3) δ15.2, 55.8, 108.1, 119.0, 123.1, 123.8, 143.6, 146.1; MSm/z: 138 (M^+^), 123,95,67.*

### Cell Cultures and Tests using Inhibitors

The murine macrophage cell lines RAW 264.7 and J774A.1 were obtained from the American Type Culture Collection (Rockville, MD). RAW 264.7 macrophages stably transfected with the NF-κB reporter gene (RAW-Blue™ cells) were purchased from InvivoGen (San Diego, CA). All cells were cultured in RPMI 1640 medium supplemented with 10% heat-inactivated fetal calf serum and 2 mM L-glutamine (all from Life Technologies, Carlsbad, CA) at 37°C in a 5% CO_2_ incubator. In the case of RAW-Blue™ cells, 100 µg/ml of zeocin was added to the medium. In tests involving sequential additions, e.g. pretreatment with inhibitors, unless otherwise stated, all reagents present in the previous step were still present during the next step.

### AlamarBlue® Assay for Cell Viability

Cells were seeded in 96-well flat-bottom plates at a density of 5000 cells in 100 µl of RPMI 1640 medium containing 10% fetal calf serum per well and incubated for 24 h at 37°C in a 5% CO_2_ incubator, then with the test samples for 24 h, when the AlamarBlue® assay was used to determine the cytotoxicity of the test samples as described by the manufacturer (AbD Serotec, Oxford).

### Ethics Statement

All animal manipulations were performed in the Laboratory Animal Center of National Defense Medical Center (Taipei, Taiwan) in accordance with the ethical rules in the NIH guide for the care and use of laboratory animals. The procedures used were approved by the Animal Care and Use Committee of National Defense Medical Center. All manipulations were performed under isoflurane anesthesia, and all efforts were made to minimize suffering. The condition of the mice did not decline significantly during the experiment and no significant signs of suffering were observed. At the end of experiments all the mice were humanely euthanized by inhalation of an overdose of isoflurane to minimize suffering.

### Animal Model and Experimental Protocol

The experiments were performed on 8-week-old female BABL/c mice purchased from the National Laboratory Animal Breeding and Research Center (Taipei, Taiwan). The mice were divided into three groups (each n = 6), which were treated with (i) 3 µg/g body weight of LPS injected intraperitoneally (i.p.), (ii) 3 µg/g body weight of LPS injected i.p. plus 30 µg/g body weight of creosol given orally 24 h before LPS, or (iii) saline injected i.p. Serum was collected 4 h after LPS administration and the mice sacrificed 24 h after LPS administration.

### Western Blotting Analysis, Enzyme-linked Immunosorbent Assay (ELISA), Nitric Oxide (NO) Inhibitory Assay, and NF-κB Reporter Assay

These were performed as described previously [33].

### Statistical Analysis

All values are the mean *±* standard deviation (SD). Data analysis involved one-way ANOVA with a subsequent Scheffé test.

## Results

### BV Decreases NO Generation, Inducible NO Synthase Expression, and IL-6 Secretion in LPS-activated Macrophages

**Figure 1:**
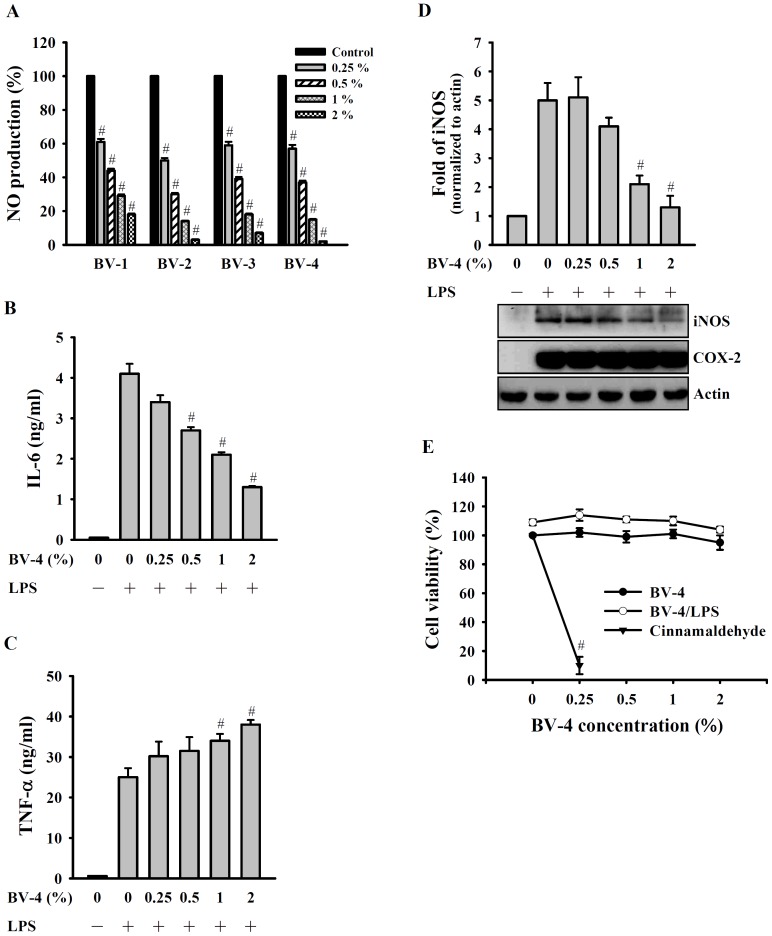
Effect of different BV samples and BV-4 on inflammatory mediator expression. In (A), RAW 264.7 macrophages (1×10^6^ in 2 ml of medium) were incubated for 30 min with or without the indicated concentrations of BV-1, BV-2, BV-3, or BV-4, then for 24 h with or without addition of 1 µg/ml of LPS, then NO generation in the culture medium was measured by the Griess reaction. In (B), (C), and (D), RAW 264.7 macrophages (1×10^6^ in 2 ml of medium) were incubated for 30 min with or without the BV-4, then for 24 h with or without addition of 1 µg/ml of LPS, then IL-6 (B) and TNF-α (C) in the culture medium were measured by ELISA and levels of iNOS and COX-2 (D) in cell lysates were measured by Western blotting. In (E), RAW 264.7 macrophages (5×10^4^ in 1 ml of medium) were incubated for 30 min with or without the indicated concentration of BV-4 or 50 µM cinnamaldehyde, then for 24 h with or without addition of 1 µg/ml of LPS, then cell viability was measured using the AlamarBlue® assay. In (A), (B), (C), and (E), the data are expressed as the mean ± SD for three separate experiments, while, in (D), the lower panel shows a typical result and the histogram shows results for 3 experiments expressed as the mean ± SD. # indicate a significant difference at the respective level of *p*<0.001 compared to the LPS-treated group.

The anti-inflammatory activity of BV produced at different temperatures (BV-1, 90–92°C; BV-2, 99–102°C; BV-3, 120–123°C; BV-4, 145–150°C) was investigated using LPS-activated RAW 264.7 macrophages. As shown in Fig. 1A, NO generation was inhibited by all BVs in a dose-dependent manner and the different BV samples had a similar effect. BV-4 was therefore used in all subsequent experiments. In the same system, we found that IL-6 secretion was inhibited by BV-4 (Fig. 1B), whereas TNF-α secretion was increased (Fig. 1C), both effects being dose-dependent. Expression of inducible NO synthase (iNOS) protein was also reduced in a dose-dependent manner by BV-4 (Fig. 1D). To examine whether the effects on NO generation, iNOS expression, and IL-6 secretion were due to reduced cell viability, the toxicity of BV-4 for RAW 264.7 macrophages was examined and BV-4 was found to have no effect on cell survival at concentrations up to at least 2% (Fig. 1E). Cinnamaldehyde was used as a positive control for reducing cell viability [34]. In addition, BV-4 was not toxic for another murine macrophage cell line, J774A.1, at concentrations up to at least 2% (data not shown).

### BV Reduces PKC-α/δ Phosphorylation in LPS-activated Macrophages

**Figure 2:**
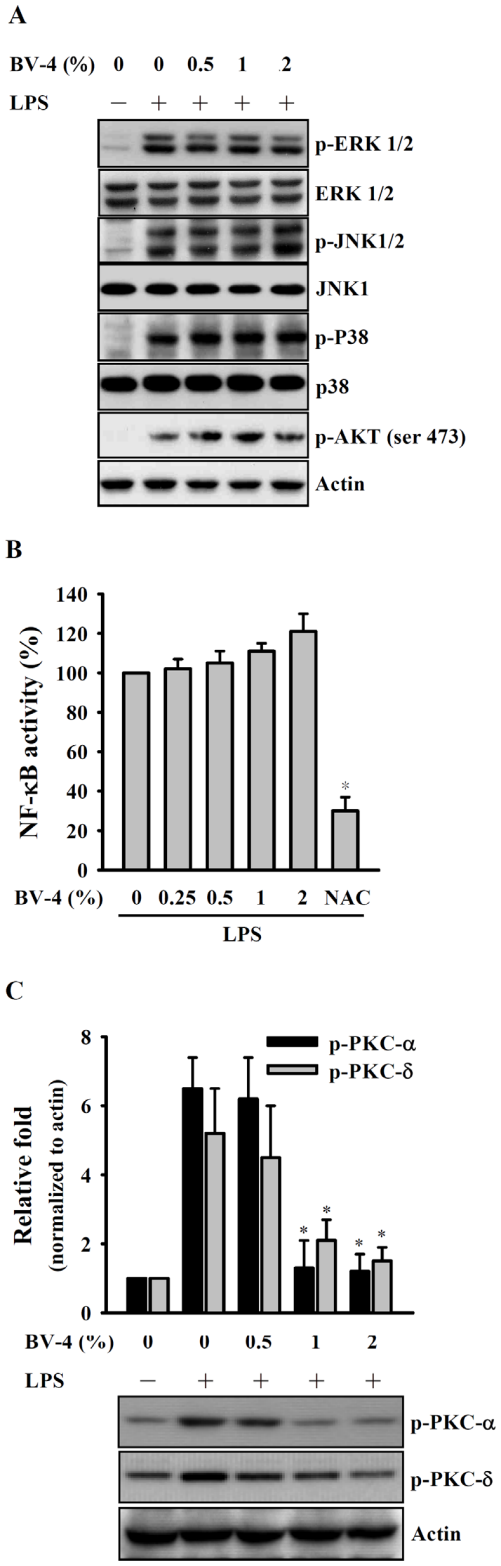
Effect of BV-4 on MAPK and PKC phosphorylation and NF-κB activation. In (A) and (C), RAW 264.7 macrophages (1×10^6^ in 2 ml of medium) were incubated for 30 min with or without the indicated concentration of BV-4, then for 20 min with or without addition of 1 µg/ml of LPS, then phosphorylation of ERK1/2, JNK1/2, p38, AKT (A) and PKC-α/δ (C) was measured by Western blotting. In (B), RAW-Blue™ cells (1×10^6^ in 2 ml of medium) were incubated for 30 min with or without the indicated concentration of BV-4, then for 24 h with or without addition of 1 µg/ml of LPS, then secreted embryonic alkaline phosphatase activity was measured using QUANTI-Blue™. In (A) and (C), the results are representative of those obtained in three different experiments and the histogram shows the results for all 3 experiments expressed as the mean ± SD, while, in (B), the data are expressed as the mean ± SD for three separate experiments. *indicates a significant difference at the level of *p*<0.05 compared to the LPS-treated group.

LPS induces macrophage activation and the production of pro-inflammatory mediators by activating TLR4 through many signaling pathways, including the MAPK, AKT, and NF-κB signaling pathways [6], [35]. Fig. 2A shows that BV-4 at concentrations of 0–2% did not affect the phosphorylation of ERK1/2, JNK1/2, p38, and AKT in LPS-activated macrophages. We then examined if BV-4 inhibited LPS-induced NO generation and IL-6 secretion by inhibiting NF-κB activation using NF-κB-dependent alkaline phosphatase reporter cells (RAW-Blue™ cells) and, as shown in Fig. 2B, found that NF-κB transcriptional activity in LPS-stimulated macrophages, rather than being decreased, was, in fact, slightly, but not significantly, increased by BV-4, although markedly inhibited by the potent antioxidant *N*-acetyl cysteine (NAC) used as the positive control. PKC is one of the components of the TLR4 signaling pathway and therefore plays a role in macrophage activation in response to LPS [36]. *As shown in* *Fig. 2C*, *the LPS-induced increase in phosphorylation of PKC-*α *and PKC-*δ* was reduced by the BV-4 in a dose-dependent manner.*

### Fractionation of BV-4

**Figure 3:**
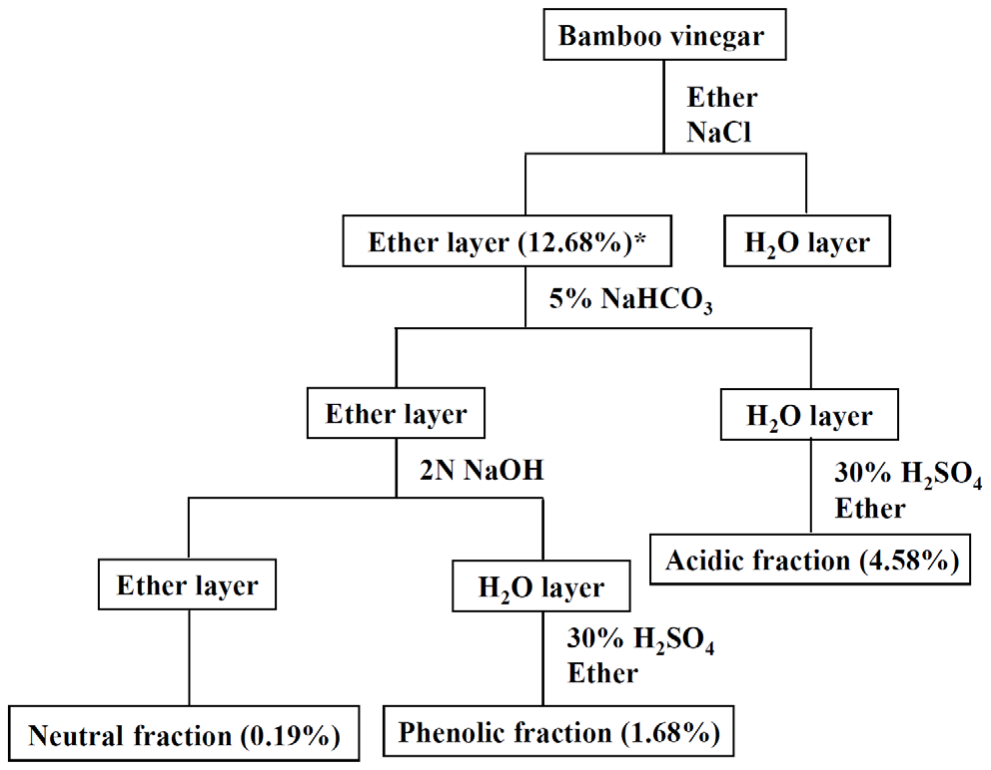
Flow chart for the fractionation of BV-4.

**Figure 4:**
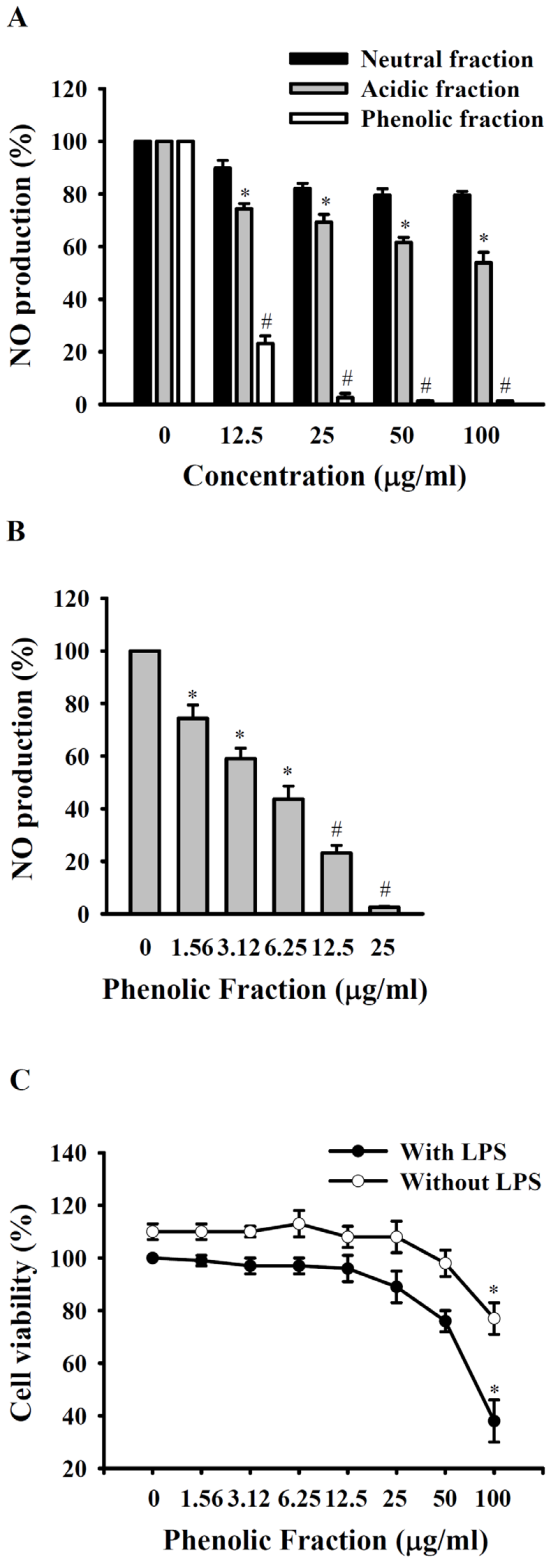
Effect of BV-4 fractions on NO generation and cell viability. In (A), RAW 264.7 macrophages (1×10^6^ in 2 ml of medium) were incubated for 30 min with or without the indicated concentrations of the neutral, acidic, or phenolic fraction of BV-4, then for 24 h with or without addition of 1 µg/ml of LPS, then NO generation in the culture medium was measured by the Griess reaction. In (B), RAW 264.7 macrophages (1×10^6^ in 2 ml of medium) were incubated for 30 min with or without the indicated concentration of the phenolic fraction of BV-4, then for 24 h with or without addition of 1 µg/ml of LPS, then NO generation in the culture medium was measured by the Griess reaction. In (C), RAW 264.7 macrophages (5×10^4^ in 1 ml of medium) were incubated for 30 min with or without the phenolic fraction of BV-4, then for 24 h with or without addition of 1 µg/ml of LPS, then cell viability was measured by the AlamarBlue® assay. The data are expressed as the mean ± SD for three separate experiments. *and # indicate a significant difference at the respective levels of *p*<0.05 and *p*<0.001 compared to the LPS-treated group.

BV-4 was fractioned into neutral, acidic, and phenolic fractions using the procedure shown in Fig. 3. The acidic fraction was the main fraction (4.58% of BV by weight), followed by the phenolic fraction (1.68%), and the neutral fraction (0.19%). As shown in Fig. 4A, the dried down phenolic fraction at concentrations of 12.5–100 µg/ml caused marked and significant inhibition of the increase in NO generation in LPS-activated macrophages, whereas a less, though significant, effect was seen with the acidic fraction and a slight and non-significant effect was seen with the neutral fraction. As shown in Fig. 4B, the inhibitory effect of the phenolic fraction was dose-dependent. Cell viability was not significantly reduced by the phenolic fraction at concentrations ≦ 50 µg/ml (Fig. 4C).

### Creosol is the Major Anti-inflammatory Compound in BV-4

**Figure 5:**
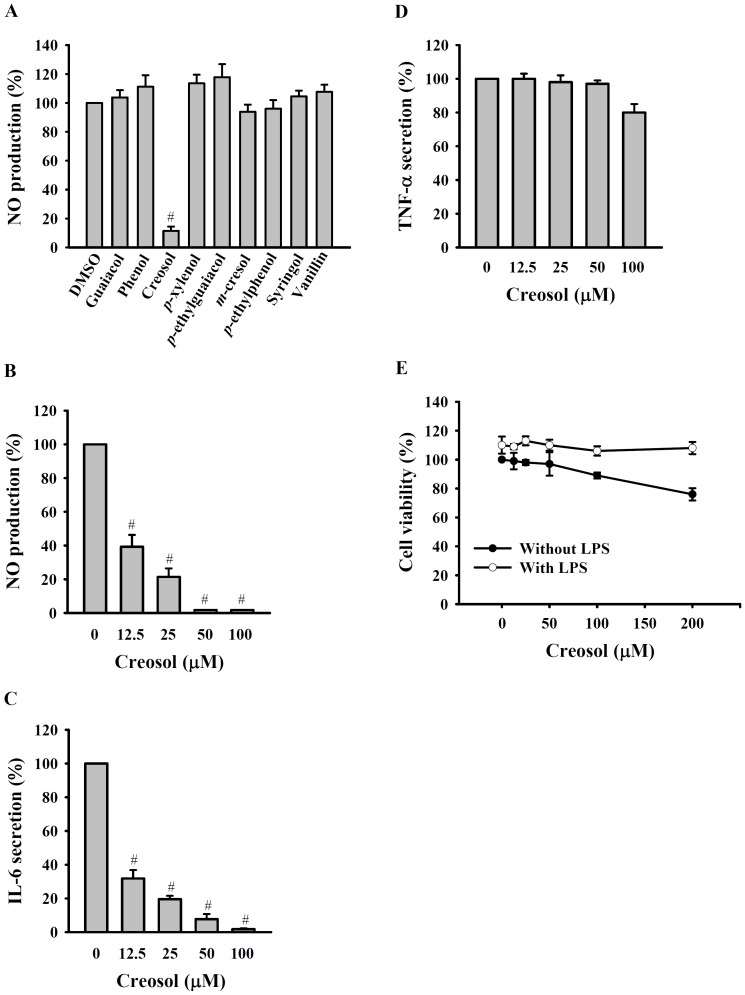
Effect of creosol on inflammatory mediator expression and cell viability. In (A), RAW 264.7 macrophages (1×10^6^ in 2 ml of medium) were incubated for 30 min with or without the test compound (50 µM), then for 24 h with or without addition of 1 µg/ml of LPS, then NO generation in the culture medium was measured by the Griess reaction. In (B), (C), and (D), RAW 264.7 macrophages (1×10^6^ in 2 ml of medium) were incubated for 30 min with or without the indicated concentration of creosol, then for 24 h with or without addition of 1 µg/ml of LPS, then NO generation in the culture medium was measured by the Griess reaction (B) and IL-6 (C) and TNF-α (D) levels in the culture medium were measured by ELISA. In (E), RAW 264.7 macrophages (5×10^4^ in 1 ml of medium) were incubated for 30 min with or without the indicated concentration of creosol, then for 24 h with or without addition of 1 µg/ml of LPS, then cell viability was measured by the AlamarBlue® assay. The data are expressed as the mean ± SD for three separate experiments. # indicates a significant difference at the respective levels of *p*<0.001 compared to the LPS-treated group.

To further characterize the chemical compositions of the acidic, neutral, and phenolic fractions of BV-4, they were analyzed by GC-MS, and the chemical compositions are shown in Table 1. To identify the active compounds in the phenolic fraction, the major compounds listed in Table 1 were evaluated for inhibition of NO generation in LPS-activated macrophages. As shown in Fig. 5A, of the components tested (all at a concentration of 50 µM), only creosol was able to reduce NO generation. The LPS-induced increase in NO generation (Fig. 5B) and IL-6 secretion (Fig. 5C) were inhibited by creosol in a dose-dependent manner, whereas TNF-α secretion was not affected (Fig. 5D). Cell viability was not significantly reduced by creosol at concentrations up to at least 200 µM (Fig. 5E).

**Table 1:** Composition of the fractions of BV-4.^a^

	Fraction (%)		
Compound	Phenolic	Acidic	Neutral
Acetic acid		84.95	
Propanoic acid		11.68	
2-Methyl-2-cyclopentenone			
Butanoic acid		0.92	
Furfural			0.60
Tetrahydrofurfuryl alcohol			
2-acetyl furan			8.41
Acetolacetate			2.22
n-Butyric anhydride			19.50
3-Furanmethanol			3.15
5-Methylfurfural			1.82
2,3-Dimethyl-2-cyclopenten-1-one			8.54
3-Methyl-2-cyclopentenone			7.95
Acetonyl acetone			2.65
Methyl 3-acetylpropanoate			5.56
3-Ethyl-2-hydroxy-2-cyclopenten-1-one			4.50
3,5-Dimethyl cyclopentenolone			3.84
3-Ethyl-2-cyclopenten-1-one	1.06		2.88
2-Furanone,2,5-dihydro-3,5-dimethyl			0.17
Guaiacol	18.92		
Valerolactone			0.66
4-Butyrolactone	0.39	0.79	5.63
3-Methyl-2(5H)-furanone			0.99
3-Ethyl-2-hydroxy-2-cyclopenten-1-one	0.06		1.66
Isocresol	0.06		
*o*-Cresol	2.72		
Phenol	25.27	0.96	
Creosol	10.72		
*o*-Ethylphenol	0.06		4.30
*p*-Xylenol	0.80		
*p*-Ethylguaiacol	4.46		3.31
*p*-Cresol	4.55		
*m*-Cresol	2.99		3.97
*p*-Ethylphenol	8.64		2.98
Syringol	17.78	0.67	
Isovanillic acid	0.90		
Vanillin	0.50		
**Total** b	**99.88**	**99.97**	**95.30**

a The numbers below are the percentage of the weight of crude vinegar found in each fraction.

b Sum of the percentages of components in each fraction.

### Creosol reduces IL-1β Secretion by Inhibiting NLRP3 Inflammasome Activation

**Figure 6:**
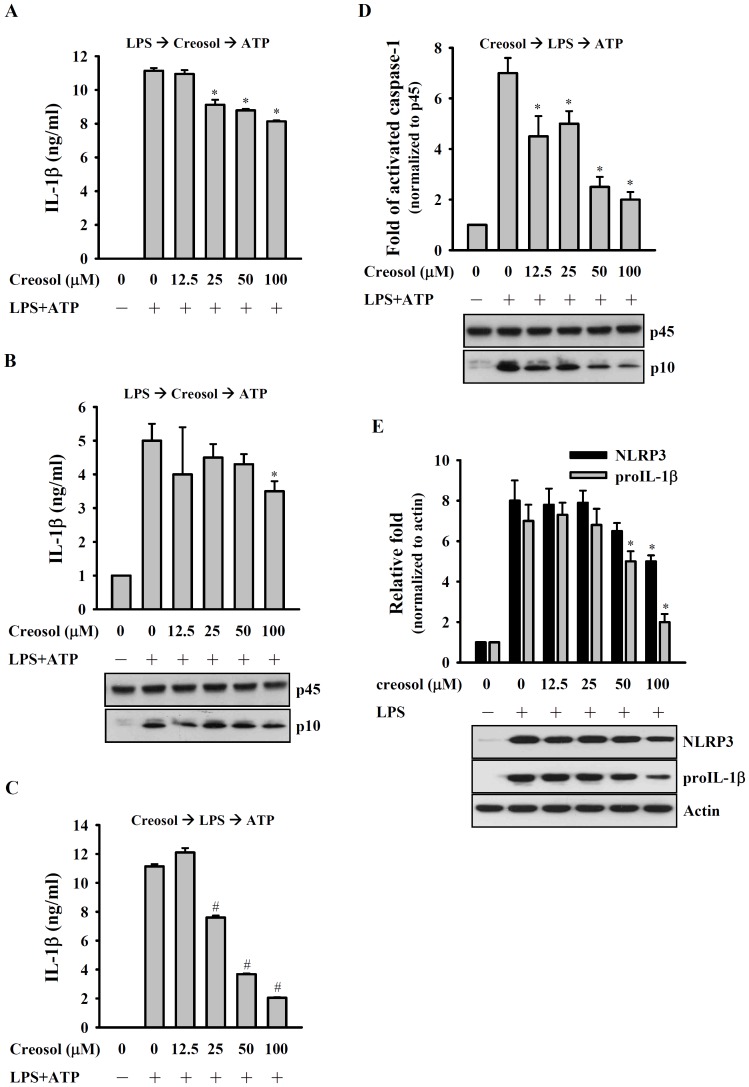
Effect of creosol on NLRP3 inflammasome activation. In (A) and (B), J774A.1 macrophages (2×10^6^ in 2 ml of medium) were incubated for 6 h with or without 1 µg/ml of LPS, then for 30 min with or without addition of the indicated concentration of creosol, followed by 30 min incubation with or without addition of 5 mM ATP, then IL-1β in the culture medium were measured by ELISA (A) and active caspase-1 (p10) and caspase-1 (p45) in the cells were measured by Western blotting (B). In (C) and (D), J774A.1 macrophages (2×10^6^ in 2 ml of medium) were incubated for 30 min with or without the indicated concentration of creosol, then for 6 h with or without addition of 1 µg/ml of LPS. After washing, the cells were incubated with or without 5 mM ATP for 30 min, then IL-1β in the culture medium was measured by ELISA (C) and active caspase-1 (p10) and caspase-1 (p45) in the cells measured by Western blotting (D). In (E), J774A.1 macrophages (2×10^6^ in 2 ml of medium) were incubated for 30 min with or without the indicated concentration of creosol and for 6 h with or without addition of 1 µg/ml of LPS, then expression of NLRP3 and proIL-1β was analyzed by Western blotting. In (A) and (C), the data are expressed as the mean ± SD for three separate experiments, while, in (B), (D), and (E), the results are representative of those obtained in three different experiments and the histogram shows the results for all 3 experiments expressed as the mean ± SD. *and # indicate a significant difference at the respective levels of *p*<0.05 and *p*<0.001 compared to the LPS+ATP-treated group (A-D) or the LPS-treated group (E).

RAW 264.7 macrophages are not suitable for studying NLRP3 inflammasome; therefore, we choose J774A.1 macrophages to study the effect of creosol on NLRP3 inflammasome. We then investigated whether creosol was able to inhibit NLRP3 inflammasome activation in J774A.1 macrophages activated by LPS and ATP [37]. ATP induced IL-1β secretion (Fig. 6A and C) and caspase-1 activation (Fig. 6B and D) in LPS-primed cells. And both effects were inhibited by pre-incubation of the cells with different concentrations of creosol before (Fig. 6C and D) or after (Fig. 6A and B) LPS stimulation. Furthermore, we investigated whether creosol was able to inhibit expression of NLRP3 protein, an essential component of the NLRP3 inflammasome, and of proIL-1β the precursor of IL-1β, in LPS-activated macrophages. As shown in Fig. 6E, the LPS-induced increase in NLRP3 and proIL-1β protein levels was inhibited by creosol. These results show that creosol inhibits NLRP3 inflammasome activation by acting on both the ATP- and LPS-mediated signaling stages.

### Creosol Inhibits LPS- and ATP-induced ROS Production

**Figure 7:**
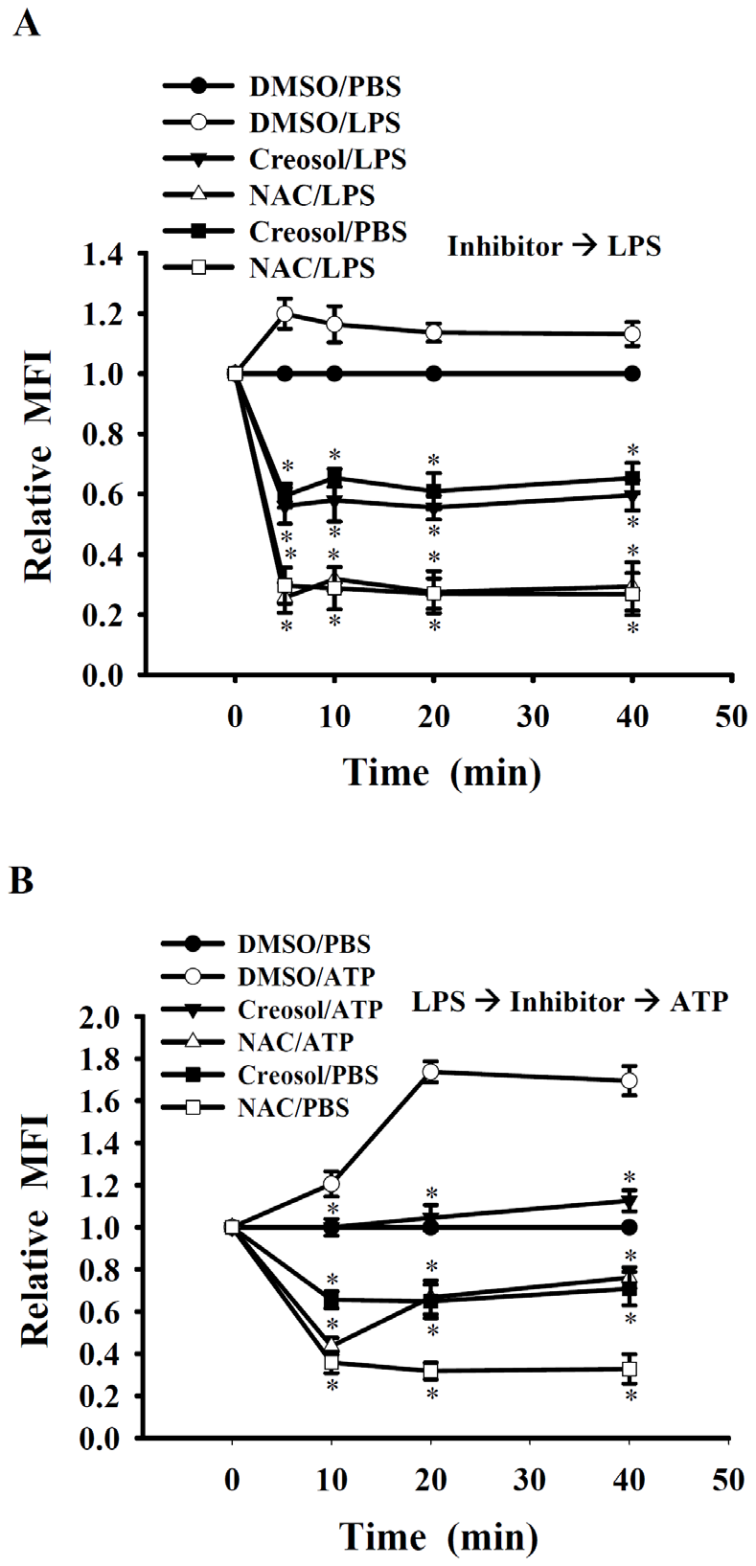
Effect of creosol on LPS- and ATP-induced ROS production. In (A), J774A.1 macrophages (1×10^6^ in 1 ml of medium) were incubated for 30 min with or without 50 µM creosol or 10 mM N-acetyl cysteine (NAC), then for 0–40 min with or without addition of 1 µg/ml of LPS. In (B), J774A.1 macrophages (1×10^6^ in 1 ml of medium) were incubated for 6 h with 1 µg/ml of LPS, then LPS was washout, then for 30 min with or without addition of 50 µM creosol or 10 mM NAC, then for 0–40 min with or without addition of 5 mM ATP. ROS production was measured as the relative mean fluorescence intensity (MFI), as described in the Materials and Methods. The data are expressed as the mean ± SD for three separate experiments. *indicates a significant difference at the level of *p*<0.05 compared to the DMSO/LPS-treated group (A) or the DMSO/ATP-treated group (B).

The LPS-induced increase in ROS production was reduced by incubation with creosol (50 µM) and the potent antioxidant NAC (10 mM) 30 min before, and during, LPS stimulation (Fig. 7A). Furthermore, the ATP-induced increase in ROS production was reduced by incubation with creosol and the NAC 30 min before, and during, ATP stimulation (Fig. 7B). These results suggest that the anti-inflammatory effect of creosol involves its anti-oxidative activity.

### Anti-inflammatory Effect of Creosol in vivo

**Figure 8:**
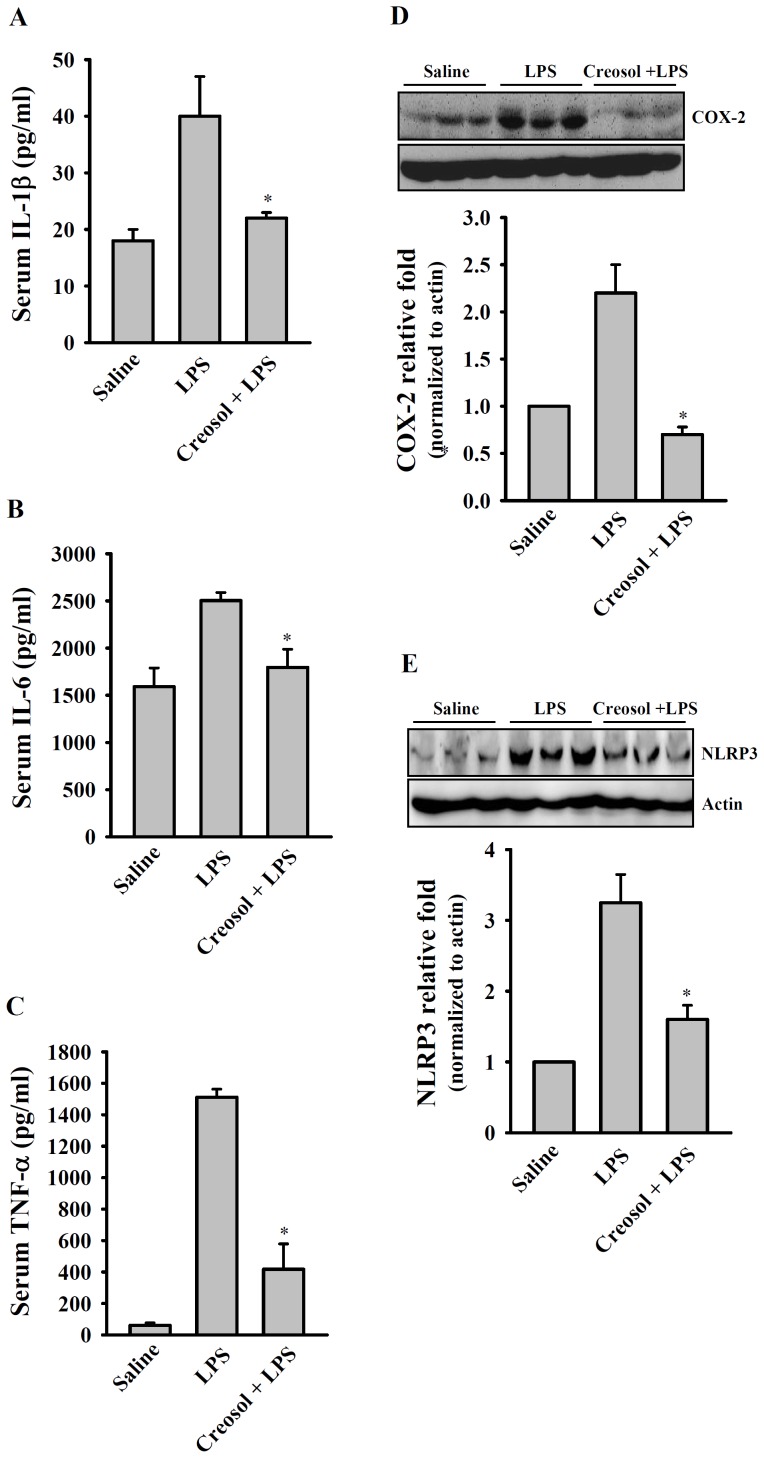
Effect of creosol on LPS-induced inflammation in vivo. Three groups of mice (n = 6 each) were treated with LPS (3 µg/g body weight, given intraperitoneally), LPS (3 µg/g body weight, given intraperitoneally) plus creosol (30 µg/g body weight, given orally 24 h before LPS), or saline alone. At 4 h after LPS injection, serum was collected and assayed for IL-1β (A), IL-6 (B), and TNF-α (C) by ELISA, and, at 24 h, the spleen and liver were collected and assayed, respectively, for expression of COX-2 (D) or NLRP3 (E) by Western blotting. In (A), (B), and (C), the data are expressed as the mean ± SD for three separate experiments, while, in (D) and (E), the results are representative of those obtained in three different experiments and the histogram shows the results for all expressed as the mean ± SD. *indicates a significant difference at the level of *p*<0.05 compared to LPS-injected mice.

Mice were left untreated or were injected with LPS with or without oral administration of creosol (30 µg/g body weight) 24 h beforehand, then serum samples were collected 4 h after LPS injection to measure levels of IL-1β, IL-6, and TNF-α and the spleen and liver were collected at 24 h after injection to measure COX-2 and NLRP3 levels. LPS injection resulted in a significant increase in serum levels of IL-1β (Fig. 8A), IL-6 (Fig. 8B), and TNF-α (Fig. 8C) compared to the saline-injected controls and these effects were markedly inhibited in the creosol-pretreated mice. The LPS-injected mice also showed a significant increase in COX-2 protein levels in the spleen (Fig. 8D) and NLRP3 protein levels in the liver (Fig. 8E) and both of these effects were significantly inhibited by creosol pretreatment.

## Discussion

Bamboos are of notable economic significance, as they are used as a construction material, in paper manufacture and water desalination, and as a food source. Bamboo is also used in Chinese and Indian traditional medicine for treating diseases. Supplementation with bamboo extract lowers serum levels of monocyte chemoattractant protein-1 in mice fed a high-fat diet [38]. Monocyte chemoattractant protein-1 is an inflammatory chemokine upregulated in obese subjects and contributes to the development of type 2 diabetes [39]. Bamboo extract also inhibits the palmitic acid-induced increase in IL-6 secretion by a mouse muscle cell line and an adipose cell line by reducing NF-κB and AP-1 activation [40]. In addition, bamboo salt has anti-inflammatory activity on a human mast cell line by reducing the increase in TNF-α, IL-1β and IL-6 expression induced by phorbol 12-myristate 13-acetate plus a calcium ionophore [41]. It also inhibits cisplatin-induced ROS production and apoptosis in a mouse auditory cell line, suggesting it may prevent the ototoxic side effects of cisplatin in patients undergoing chemotherapy [42]. Tricin, isolated from bamboo leaves, is considered sufficiently safe to undergo clinical development as a cancer chemopreventive agent [43]. Although many medicinal applications of bamboo and its products have been reported, little is known about the medicinal applications of BV.

In the present study we demonstrated that BV exhibited anti-inflammatory activity by reducing NO generation and IL-6 secretion in LPS-activated macrophages. It is now evident that there is a strong link between NO and the progression of Alzheimer’s disease and reducing NO generation is a goal in the treatment of this disease [44]. Reduction of IL-6 expression might prevent or ameliorate the pathogenesis of cancer [45], type 2 diabetes, and cardiovascular disease [46]. The p38 and NF-κB pathways play important roles in NO generation in LPS-activated macrophages [47]; however, BV-4 did not inhibit these pathways, although it reduced NO generation. BV-4 did inhibit PKC-α/δ activation and PKC-α/δ play important roles in NO generation [47]. Creosol was found to be the major anti-inflammatory compound in the phenolic fraction of BV, and was able to reduce not only conventional inflammatory responses, such as NO generation and IL-6 secretion in LPS activated macrophages, but also NLRP3 inflammasome-mediated IL-1β expression in LPS- and ATP-activated macrophages. It inhibited caspase-1 activation and IL-1β secretion when added before or after LPS treatment, indicating that it inhibits both the priming and activation signals for NLRP3 inflammasome in LPS- and ATP-activated macrophages, although it was more potent when added before LPS compared to after. This result might be explained by the observations that creosol not only reduced caspase-1 activation, but also inhibited expression of NLRP3 and proIL-1β in LPS-primed cells. Recently, NLRP3 inflammasome has become an important target for healthy/or functional foods. For instance, Chinese herb *Hirsutella sinensis* mycelium extracts exhibit anti-inflammatory activity by inhibiting NLRP3 inflammasome [48]. *In the previous studies we demonstrated that antroquinonol, a pure compound from medical fungus Antrodia camphorata* mycelium, and Epigallocatechin-3-gallate, a pure compound from green tea, ameliorates the progression of IgA nephropathy and IgA nephropathy respectively by inhibiting NLRP3 inflammasome [49], [50]. In the other study, the anti-tumorigenic mushroom *Agaricus blazei* Murill extracts induce IL-1β secretion through NLRP3 inflammasome [51]. The creosol concentration in each of the BV samples produced at different temperatures was similar (data not shown), explaining why each BV had a similar potency in NO inhibition. Prolonged and acute inflammation characterized by excessive production of inflammatory mediators can be harmful because it may cause host toxicity and tissue damage; however, inflammatory responses for a short and controlled duration can be beneficial because they help against the infection. Although BV reduced IL-1β, IL-6, and NO production in LPS-activated macrophages, it increased TNF-α secretion significantly and NF-κB activation slightly, but not significantly at high concentration (1–2%). These results suggest that BV modulates immune responses, but does not cause overall immune suppression in host during infection.

ROS play a pivotal role in LPS-mediated NO generation and IL-6 secretion by macrophages [33] and in NLRP3 and proIL-1β expression in macrophages [37]. In the present study, we demonstrated that creosol decreased both the LPS- and ATP-mediated steps in ROS generation, showing that creosol has anti-oxidant activity. ERK1/2, JNK1/2, and p38 play important roles in LPS-induced pro-inflammatory responses [33]. These kinases were not inhibited by the BV-4; however, the detailed mechanism is not clear yet. We believed that this anti-oxidant activity of creosol and BV is important for their anti-inflammatory activity, especially inhibition of NLRP3 inflammasome activation. Previous reports have shown that creosol can prevent the death of cultured rat hippocampal neurons exposed to N-methyl-D-aspartate or H_2_O_2_ by reducing both Ca^2+^ influx and the generation of intracellular ROS [52], [53]. Creosol also prevents ovariectomy-induced bone loss by inhibiting osteoclastogenesis and its anti-oxidative effect on osteoblasts [54].

It is well-known that incomplete combustion of organic matter produces a wide range of carcinogenic compounds, such as the polycyclic aromatic hydrocarbons found in cigarette smoke, car fuel exhaust, and BBQ-cooked foods. BV, produced during bamboo charcoal production, is not traditionally used for medical and food applications, so the safety and possible carcinogenicity of BV should be considered before it is used medicinally. When the carcinogenic and tumor-promoting potential of BV was examined in the *in vitro* BALB/c 3T3 A31-1-1 cell transformation assay system, BV was found not to act as a tumor promoter [55]. In addition, it has been reported that increasing single doses of wood creosote, a mixture of creosol, guaiacol, and related compounds with a similar composition to BV, were safe and well tolerated in healthy men and women and that the wood creosote was rapidly absorbed, conjugated, and eliminated [56], [57]. However, the cited studies were performed with diluted samples and possibly with only a single exposure. We can’t rule out the possibility yet that BV may actually have carcinogenic potential at higher concentration or after prolonged use. Creosol, also a volatile compound, is found in wines matured in oak barrels [58]. Furthermore, it is one of the major ingredients of a traditional antidiarrheic drug that has been used for more than 100 years in Japan [54]. In this study we demonstrated that the concentrations of BV or creosol required for anti-inflammatory activity were non-toxic for macrophages. The composition of the fractions of BV was assayed by GC-MS; however, this approach may identify only the volatile compounds present in the extract and the other important active compounds may have been overlooked. The other analysis methods, such as HPLC and NMR, could be applied for further analysis.

In summary, we have demonstrated that BV and its active component, creosol, have anti-inflammatory activity by reducing NO levels, IL-6 secretion, and NLRP3 inflammasome activation. The anti-inflammatory activity of BV and creosol is associated with their anti-oxidant and PKC inhibitory activity. These results suggest that BV may have potential for use as a natural and cost-effective anti-inflammation agent or nutraceutical for preventing and ameliorating inflammation- and NLRP3 inflammasome-related diseases, including metabolic syndrome.

## References

[1] Akakabe Y, Tamura Y, Iwamoto S, Takabayashi M, Nyuugaku T (2006) Volatile organic compounds with characteristic odor in bamboo vinegar. Biosci Biotechnol Biochem 70 2797–2799. 17090925 10.1271/bbb.60317

[2] Chen YX, Huang XD, Han ZY, Huang X, Hu B et al (2010) Effects of bamboo charcoal and bamboo vinegar on nitrogen conservation and heavy metals immobility during pig manure composting. Chemosphere 78 1177–1181. 20060567 10.1016/j.chemosphere.2009.12.029

[3] Ruttanavut J, Matsumoto Y, Yamauchi K (2012) A fluorescence-based demonstration of intestinal villi and epithelial cell in chickens fed dietary silicic acid powder including bamboo vinegar compound liquid. Histol Histopathol 27 1333–1342. 22936452 10.14670/HH-27.1333

[4] Marumoto S, Yamamoto SP, Nishimura H, Onomoto K, Yatagai M et al (2012) Identification of a germicidal compound against picornavirus in bamboo pyroligneous acid. J Agric Food Chem 60 9106–9111. 22849724 10.1021/jf3021317

[5] Medzhitov R, Janeway CA (1997) Innate immunity: the virtues of a nonclonal system of recognition. Cell 91 295–298. 9363937 10.1016/s0092-8674(00)80412-2

[6] Takeda K, Kaisho T, Akira S (2003) Toll-like receptors. Annu Rev Immunol 21 335–376. 12524386 10.1146/annurev.immunol.21.120601.141126

[7] Schroder K, Tschopp J (2010) The inflammasomes. Cell 140 821–832. 20303873 10.1016/j.cell.2010.01.040

[8] Davis BK, Wen H, Ting JP (2011) The inflammasome NLRs in immunity, inflammation, and associated diseases. Annu Rev Immunol 29 707–735. 21219188 10.1146/annurev-immunol-031210-101405 PMC4067317

[9] Cassel SL, Joly S, Sutterwala FS (2009) The NLRP3 inflammasome: a sensor of immune danger signals. Semin Immunol 21 194–198. 19501527 10.1016/j.smim.2009.05.002 PMC2758520

[10] Jin C, Flavell RA (2010) Molecular mechanism of NLRP3 inflammasome activation. J Clin Immunol 30 628–631. 20589420 10.1007/s10875-010-9440-3

[11] Allen IC, Scull MA, Moore CB, Holl EK, McElvania TE et al (2009) The NLRP3 inflammasome mediates in vivo innate immunity to influenza A virus through recognition of viral RNA. Immunity 30 556–565. 19362020 10.1016/j.immuni.2009.02.005 PMC2803103

[12] Gross O, Poeck H, Bscheider M, Dostert C, Hannesschläger N et al (2009) Syk kinase signalling couples to the Nlrp3 inflammasome for anti-fungal host defence. Nature 459 433–436. 19339971 10.1038/nature07965

[13] Martinon F, Pétrilli V, Mayor A, Tardivel A, Tschopp J (2006) Gout-associated uric acid crystals activate the NALP3 inflammasome. Nature 440 237–241. 16407889 10.1038/nature04516

[14] Duewell P, Kono H, Rayner KJ, Sirois CM, Vladimer G et al (2010) NLRP3 inflammasomes are required for atherogenesis and activated by cholesterol crystals. Nature 464 1357–1361. 20428172 10.1038/nature08938 PMC2946640

[15] Vandanmagsar B, Youm YH, Ravussin A, Galgani JE, Stadler K et al (2011) The NLRP3 inflammasome instigates obesity-induced inflammation and insulin resistance. Nat Med 17 179–188. 21217695 10.1038/nm.2279 PMC3076025

[16] Halle A, Hornung V, Petzold GC, Stewart CR, Monks BG et al (2008) The NALP3 inflammasome is involved in the innate immune response to amyloid-beta. Nat Immunol 9 57–65. 10.1038/ni.1636 PMC3101478 18604209

[17] Hornung V, Bauernfeind F, Halle A, Samstad EO, Kono H et al (2008) Silica crystals and aluminum salts activate the NALP3 inflammasome through phagosomal destabilization. Nat Immunol 9 847–856. 18604214 10.1038/ni.1631 PMC2834784

[18] Bauernfeind FG, Horvath G, Stutz A, Alnemri ES, MacDonald K et al (2009) Cutting edge: NF-kappaB activating pattern recognition and cytokine receptors license NLRP3 inflammasome activation by regulating NLRP3 expression. J Immunol 183 787–791. 19570822 10.4049/jimmunol.0901363 PMC2824855

[19] Bauernfeind F, Bartok E, Rieger A, Franchi L, Núñez G et al (2011) Cutting edge: Reactive oxygen species inhibitors block priming, but not activation, of the NLRP3 inflammasome. J Immunol 187 613–617. 21677136 10.4049/jimmunol.1100613 PMC3131480

[20] Hsu HY, Wen MH (2002) Lipopolysaccharide-mediated reactive oxygen species and signal transduction in the regulation of interleukin-1 gene expression. J Biol Chem 277 22131–22139. 11940570 10.1074/jbc.M111883200

[21] Tschopp J, Schroder K (2010) NLRP3 inflammasome activation: The convergence of multiple signalling pathways on ROS production?. Nat Rev Immunol 10 210–215. 20168318 10.1038/nri2725

[22] Saïd SN, Padilla E, Langsley G, Ojcius DM (2010) Aspergillus fumigatus stimulates the NLRP3 inflammasome through a pathway requiring ROS production and the Syk tyrosine kinase. PLoS One 5 e10008. 20368800 10.1371/journal.pone.0010008 PMC2848854

[23] Anders HJ, Muruve DA (2011) The inflammasomes in kidney disease. J Am Soc Nephrol 22 1007–1018. 21566058 10.1681/ASN.2010080798

[24] Schroder K, Zhou R, Tschopp J (2010) The NLRP3 inflammasome: a sensor for metabolic danger?. Science 327 296–300. 20075245 10.1126/science.1184003

[25] Okamoto M, Liu W, Luo Y, Tanaka A, Cai X et al (2010) Constitutively active inflammasome in human melanoma cells mediating autoinflammation via caspase-1 processing and secretion of interleukin-1beta. J Biol Chem 285 6477–6488. 20038581 10.1074/jbc.M109.064907 PMC2825443

[26] Lin HC, Ohuchi T, Murase Y, Shiah TC, Shieh SL et al (2006) Application of bamboo vinegar with vacuum process to evaluate fungi resistance of bamboo materials. Journal of the Faculty of Agriculture Kyushu University. Japan 51 5–11.

[27] Lin HC, Shiah TC (2006) Evaluation of fungi resistance of moso bamboo materials using bamboo vinegar with smoking process. Quart Journal Forest Research of Taiwan. Taiwan ROC 28 51–66.

[28] Shiah TC, Wu SK, Huang JC, Lin HC (2006) The fungi resistance of bamboo materials treated with bamboo vinegar using soaking treatment. Journal of Agriculture and Forestry, NCYU 3 1–22.

[29] Uchimura T, Tanikai H, Hosoukawa K (2000) Issues of bamboo charcoal and bamboo vinegar. Soumorisya Publication, 138–168.

[30] Jodai S, Yano S, Uehara T (1989) Components of wood-vinegar liquors and their smoke flavors (in Japanese). Mokuzai Gakkaishi 35 555–563.

[31] Momose T (1997) Organic analysis. Hirokawa, Tokyo, 147.

[32] Mclafferty FW, Stauffer DB (1989) The Wiley/NBS registry of mass spectral data. Wiley, New York.

[33] Liao PC, Chien SC, Ho CL, Wang EI, Lee SC et al (2010) Osthole regulates inflammatory mediator expression through modulating NF-κB, mitogen-activated protein kinases, protein kinase C, and reactive oxygen species. J Agric Food Chem 58 10445–10451. 20839800 10.1021/jf102812t

[34] Chao LK, Hua KF, Hsu HY, Cheng SS, Lin IF et al (2008) Cinnamaldehyde inhibits pro-inflammatory cytokines secretion from monocytes/macrophages through suppression of intracellular signaling. Food Chem Toxicol 46 220–231. 17868967 10.1016/j.fct.2007.07.016

[35] Joung SM, Park ZY, Rani S, Takeuchi O, Akira S et al (2011) Akt contributes to activation of the TRIF-dependent signaling pathways of TLRs by interacting with TANK-binding kinase 1. J Immunol 186 499–507. 21106850 10.4049/jimmunol.0903534

[36] Su SC, Hua KF, Lee H, Chao LK, Tan SK et al (2006) LTA and LPS mediated activation of protein kinases in the regulation of inflammatory cytokines expression in macrophages. Clin Chim Acta 374 106–115. 16899235 10.1016/j.cca.2006.05.045

[37] Liao PC, Chao LK, Chou JC, Dong WC, Lin CN et al (2013) Lipopolysaccharide/adenosine triphosphate-mediated signal transduction in the regulation of NLRP3 protein expression and caspase-1-mediated interleukin-1β secretion. Inflamm Res 62 89–96. 22986467 10.1007/s00011-012-0555-2

[38] Higa JK, Liu W, Berry MJ, Panee J (2010) Supplement of bamboo extract lowers serum monocyte chemoattractant protein-1 concentration in mice fed a diet containing a high level of saturated fat. Br J Nutr 106 1810–1813. 10.1017/S0007114511002157 PMC4659480 21736779

[39] Panee J (2012) Monocyte Chemoattractant Protein 1 (MCP-1) in obesity and diabetes. Cytokine 60 1–12. 22766373 10.1016/j.cyto.2012.06.018 PMC3437929

[40] Higa JK, Panee J (2011) Bamboo extract reduces interleukin 6 (IL-6) overproduction under lipotoxic conditions through inhibiting the activation of NF-κB and AP-1 pathways. Cytokine 55 18–23. 21474329 10.1016/j.cyto.2011.02.019 PMC3104063

[41] Shin HY, Lee EH, Kim CY, Shin TY, Kim SD et al (2003) Anti-inflammatory activity of Korean folk medicine purple bamboo salt. Immunopharmacol Immunotoxicol 25 377–384. 19180800 10.1081/iph-120024505

[42] Jeong HJ, Kim JJ, Kim MH, Kim HM (2011) Specific blockage of caspase-1 activation by purple bamboo-salt prevents apoptosis of auditory cell line, HEI-OC1. J Med Food 14 53–61. 21128834 10.1089/jmf.2010.1232

[43] Jiao J, Zhang Y, Liu C, Liu J, Wu X et al (2007) Separation and purification of tricin from an antioxidant product derived from bamboo leaves. J Agric Food Chem 55 10086–10092. 18001030 10.1021/jf0716533

[44] Fernandez AP, Pozo RA, Serrano J, Martinez MR (2010) Nitric oxide: target for therapeutic strategies in Alzheimer’s disease. Curr Pharm Des 16 2837–2850. 20698819 10.2174/138161210793176590

[45] Waldner MJ, Foersch S, Neurath MF (2012) Interleukin-6–a key regulator of colorectal cancer development. Int J Biol Sci 8 1248–1253. 23136553 10.7150/ijbs.4614 PMC3491448

[46] Li L, Wu LL (2012) Adiponectin and interleukin-6 in inflammation-associated disease. Vitam Horm 90 375–395. 23017723 10.1016/B978-0-12-398313-8.00014-2

[47] Hua KF, Wang SH, Dong WC, Lin CY, Ho CL et al (2012) High glucose increases nitric oxide generation in lipopolysaccharide-activated macrophages by enhancing activity of protein kinase C-α/δ and NF-κB. Inflamm Res 61 1107–1116. 22706318 10.1007/s00011-012-0503-1

[48] Huang TT, Chong KY, Ojcius DM, Wu YH, Ko YF et al (2013) Hirsutella sinensis mycelium suppresses interleukin-1β and interleukin-18 secretion by inhibiting both canonical and non-canonical inflammasomes. Sci Rep 3 1374. 23459183 10.1038/srep01374 PMC3587886

[49] Yang SM, Ka SM, Hua KF, Wu TH, Chuang YP et al (2013) Antroquinonol mitigates an accelerated and progressive IgA nephropathy model in mice by activating the Nrf2 pathway and inhibiting T cells and NLRP3 inflammasome. Free Radic Biol Med 61C 285–297. 10.1016/j.freeradbiomed.2013.03.024 23567192

[50] Tsai PY, Ka SM, Chang JM, Chen HC, Shui HA et al (2011) Epigallocatechin-3-gallate prevents lupus nephritis development in mice via enhancing the Nrf2 antioxidant pathway and inhibiting NLRP3 inflammasome activation. Free Radic Biol Med 51(3) 744–754. 21641991 10.1016/j.freeradbiomed.2011.05.016

[51] Huang TT (2012) Ojcius DM, Young JD, Wu YH, Ko YF et al (2012) The anti-tumorigenic mushroom Agaricus blazei Murill enhances IL-1β production and activates the NLRP3 inflammasome in human macrophages. PLoS One 7(7) e41383. 22844468 10.1371/journal.pone.0041383 PMC3402498

[52] Matsushima N, Nakamichi N, Kambe Y, Takano K, Moriguchi N et al (2007) Cytoprotective properties of phenolic antidiarrheic ingredients in cultured astrocytes and neurons of rat brains. Eur J Pharmacol 567 59–66. 17475240 10.1016/j.ejphar.2007.03.034

[53] Nakamichi N, Fukumori R, Takarada T, Kambe Y, Yamamoto T et al (2010) Preferential inhibition by antidiarrheic 2-methoxy-4-methylphenol of Ca(2+) influx across acquired N-methyl-D-aspartate receptor channels composed of NR1/NR2B subunit assembly. J Neurosci Res 88 2483–2493. 20623618 10.1002/jnr.22399

[54] Moriguchi N, Hinoi E, Takarada T, Matsushima N, Uno K et al (2007) Oral administration of phenolic antidiarrheic ingredients prevents ovariectomy-induced bone loss. Biochem Pharmacol 73 385–393. 17078932 10.1016/j.bcp.2006.09.025

[55] Kimura Y, Suto S, Tatsuka M (2002) Evaluation of carcinogenic/co-carcinogenic activity of chikusaku-eki, a bamboo charcoal by-product used as a folk remedy, in BALB/c 3T3 cells. Biol Pharm Bull 25 1026–1029. 12186403 10.1248/bpb.25.1026

[56] Ogata N, Matsushima N, Shibata T (1995) Pharmacokinetics of wood creosote: glucuronic acid and sulfate conjugation of phenolic compounds. Pharmacology 51 195–204. 7501706 10.1159/000139335

[57] Kuge T, Shibata T, Willett MS (2003) Wood creosote, the principal active ingredient of seirogan, an herbal antidiarrheal medicine: a single-dose, dose-escalation safety and pharmacokinetic study. Pharmacotherapy 23 1391–1400. 14620385 10.1592/phco.23.14.1391.31940

[58] Pérez PLJ, López RJM, Martínez CA, Pardo MF, Gómez PE (2003) Extraction and formation dynamic of oak-related volatile compounds from different volume barrels to wine and their behavior during bottle storage. J Agric Food Chem 51 5444–5449. 12926895 10.1021/jf0345292

